# The Sanitary Concerns of the British Army

**Published:** 1855-09

**Authors:** John Davy


### ORIGINAL COMMUNICATIONS.

### THE SANITARY CONCERNS OF THE BRITISH ARMY.

By John Davy, M.D., F.R.S.

Great good surely will result from the discussion of this subject, if carried on as it has been commenced in the first number of this Journal, in the article entitled "Sanitary Regulations in the British Army,"—an article, I am free to say, at once truthful, spirited, and patriotic.

How much might be done for the advancement of medical science, and how much in return might medical science do for the public service in promoting the health and efficiency of the army, were the medical department of the army properly organized, in its administrative part, or that which is commonly called the Army Medical Board; and had that board authority from government for carrying into effect the measures which science points out as requisite; measures, the neglect of which hitherto has been the cause of so many losses, and of so much human suffering and mortality.

How should the medical board be constituted to render it what the wants of the public service require? What is the authority it ought to be allowed to possess to render it as useful and as efficient as possible?

As to the first question, ought it not to be composed of men of ample experience, of broad scientific attainments, of strict integrity; their experience acquired in the service, their attainments shown by their contributions to science, their integrity tried and proved in the same service. And ought not the appointment to be held out as the reward of merit, and made an object of honourable ambition?

As to the second question, the delegated authority, ought it not to be ample, with a responsibility corresponding? Ought not all things coming under the health-concerns of the army to be regulated by this board? as the clothing of the troops, their rations, and the construction of barracks and hospitals.

In regard to these things, how stands the case at present? Briefly as follows: In the board for "the inspection and regulation of the clothing of the army", there is no medical officer; and, as we know full well, till very lately, the health of the troops has been the least thing considered in fixing on either the form or quality of the soldier's dress. The like neglect has been shown as regards the diet of the men; instead of his ration having been fixed by medical authority, with few exceptions all the suggestions which have been offered by medical officers on the subject of the soldier's diet have either received no attention, or have been only partially acted on. In the instance of the construction of hospital and barrack buildings, we have proof of the like inattention in the appeal recently made to the Minister of War in the form of a memorial (see the preceding number of this Journal) signed by medical men, many of them medical officers of long experience in the army and navy.

It is lamentable to reflect—and I speak from painful experience—on the direful results, both in time of peace and war, the consequences of these various neglects. How many an hospital and barrack in our colonies has been built in unhealthy situations, and some even in opposition to the advice tendered by medical officers! And how ill is the structure of most of them, especially of the hospitals, for the objects for which they were intended, and all this mainly from the engineer having unlimited power to build them as he may think proper, without even consulting the medical officer on the station! It would be tedious, and it is now perhaps unnecessary, to describe even in the briefest manner the disastrous consequences of unsuitable clothing and rations. Last winter's experience before Sebastopol is a most striking and concentrated epitome of these errors.

Allusion has been made in the beginning of these remarks to the benefit that might accrue to science were the medical board properly organized; were it composed of competent men, qualified in the manner mentioned. Then, it would not fail to propose various inquiries, such as the officers of the department would have the means of engaging in; and in carrying out which an opportunity would be offered for the display of zeal, talent and attainment. In such subjects as quarantine, plague, cholera, yellow fever, tuberculosis, dysentery, what scope there is in the *questiones vexatæ* connected with them for much research! How small, considering the opportunities, has been the amount accomplished by medical officers in the public services in solving any of these questions; and how little has been effected by them in the cultivation of medical topography and meteorology, subjects specially inviting their attention, and of such great interest and importance! Nor is this surprising, when it is considered that really nothing has been done to promote such inquiries; too often, indeed, the engaging in them has hitherto been, as in civil life, an impediment to the advancement of the individuals who may have made the attempt, few as those attempts have been and far between.

In the article already alluded to, that on "The Sanitary Regulations of the British Army," a comparison has been made between the medical service of the French and English armies, much to the discredit of the latter; but, as regards inferior efficiency in the instance of the latter, not as I think altogether justly, the main difference in the two services being the possession of greater authority and power by the one than by the other. Of what avail is knowledge, is science, practically, if it cannot be brought into action? Of what avail is it to know the best sites for hospitals, the readiest means for building temporary hospitals; the simplest and easiest plans for conveying wounded men; the number of men that can be tolerated in a ward; the clothing best suited to the constitution of the soldier at home and abroad; the diets which may be most easily prepared and be most suitable under various conditions; and the amount of physical toil which is compatible with the steady enjoyment of health; of what avail, I repeat, is all this necessary knowledge, so well pointed out, if the authority necessary for carrying it into effect be wanting? Had the attainment of such knowledge been encouraged (we believe it to be possessed by many officers, gained in the service), and had the exercise of it been allowed without let or hindrance, how different would have been the history of our campaigns! In our navy we have an illustrative example in point, since we find that there by good and scientific arrangements, directed by provident foresight, the health of the crews of ships may be preserved even on the most unhealthy stations in the world, such as the western coast of Africa.

Reflecting on what was brought to light by Mr. Roebuck's committee, partial as were the disclosures made and imperfect the scrutiny, how instructive would be an inquiry, similarly instituted, regarding the health of the navy during the last winter in the Black Sea, in all respects, as far as we know, unaltered, whilst our army before Sebastopol, within sight of that fleet, owing to privations of every kind, was almost disabled—sustaining losses from death by disease manyfold greater than from the Russian fire; and that disease, be it remembered, much of the same kind as crippled our squadrons when their sanitary condition was similarly neglected in days long past.

Another desideratum I am tempted to allude to, is the fuller information that is required respecting the neglected and disastrous state of our troops in the Crimea during the past winter,—information which we know the government can furnish in the replies made by the medical officers to the questions proposed by Sir John M'Neill, when acting as commissioner of inquiry on this very subject. I have now before me a copy of a single document of the kind, drawn up by a surgeon of a regiment who was present the whole time, and very valuable it is. In acknowledging its receipt, and in exhorting him to continue the record, I thought it well to quote a passage from the article in the Journal of Public Health, already more than once referred to, where the writer describes, in reference to the catastrophe of last year, "how the whole picture was made up in fact of a grand series of physiological phenomena, arising mainly from starvation, which, if observed and recorded faithfully by any competent man, would form a most valuable contribution to the science and practice of medicine."

*Lesketh House, Ambleside,*
*August* 27, 1855.

